# Monte Carlo study of scattered and random coincidences for MADPET-4

**DOI:** 10.1186/2197-7364-2-S1-A57

**Published:** 2015-05-18

**Authors:** Negar Omidvari, Jorge Cabello, Florian Schneider, Stephan Paul, Sibyle I Ziegler

**Affiliations:** Nuklearmedizinische Klinik und Poliklinik, Klinikum rechts der Isar, Technische Universität München, Munich, Germany; Physik Department E18, Technische Universität München, Garching, ermany

MADPET-4 is a high resolution PET insert under development for use in a 7-T MR. To fully exploit the capabilities of the insert, a good understanding of the physical interactions, which take place in the active and passive components of the insert, is necessary. The goal of this study was to investigate the effects of different physical interactions in an accurate model of MADPET-4 using Monte Carlo (MC) simulations. The main focus of the study was on the impact of the different active and passive components of the system on the amount of random and scattered events, including scattering in the passive components and between the crystals. The influence of the low energy threshold (50-350 keV) and different geometrical conditions in the coincidence sorting process was of particular interest. The effect of including triple coincidences was also considered in the present study. Results showed that the maximum sensitivity achieved at the minimum energy threshold (50 keV), using a geometrical condition of 4-9 sectors, was ~3.43% without passive components and dropping to ~1.36% including the passive components. Interestingly, the minimum noise ratio was obtained with a geometrical condition of 9 sectors difference at an energy threshold of 50 keV. Including triple coincidences showed no benefit for energy thresholds above 250 keV. However, low energy thresholds provided a relative gain in sensitivity, reaching 15-20% for 200 keV and a maximum of 30-40% at 50 keV.

